# Approaching Otolaryngology Patients During the COVID-19 Pandemic

**DOI:** 10.1177/0194599820926144

**Published:** 2020-05-12

**Authors:** Chong Cui, Qi Yao, Di Zhang, Yu Zhao, Kun Zhang, Eric Nisenbaum, Pengyu Cao, Keqing Zhao, Xiaolong Huang, Dewen Leng, Chunhan Liu, Ning Li, Yan Luo, Bing Chen, Roy Casiano, Donald Weed, Zoukaa Sargi, Fred Telischi, Hongzhou Lu, James C. Denneny, Yilai Shu, Xuezhong Liu

**Affiliations:** 1 ENT Institute and Otorhinolaryngology Department of the Affiliated Eye and ENT Hospital, State Key Laboratory of Medical Neurobiology, Institutes of Biomedical Sciences, Fudan University, Shanghai, China; 2 NHC Key Laboratory of Hearing Medicine, Fudan University, Shanghai, China; 3 Department of Otorhinolaryngology, Chinese and Western Medicine Hospital of Tongji Medical College, Huazhong University of Science and Technology, Wuhan, China; 4 Department of Otolaryngology, The Third People’s Hospital of Shenzhen, Longgang District, Shenzhen, China; 5 Department of Otolaryngology, University of Miami Miller School of Medicine, Miami, Florida, USA; 6 Department of Infectious Diseases, Shanghai Public Health Clinical Center, Shanghai, China; 7 Department of Infectious Disease, Huashan Hospital, Fudan University, Shanghai, China; 8 Department of Hospital-Acquired Infection Control, Eye and ENT Hospital, Fudan University, Shanghai, China; 9 American Academy of Otolaryngology–Head and Neck Surgery, Alexandria, Virginia, USA

**Keywords:** COVID-19, SARS-CoV-2, personal protective equipment (PPE), health care worker (HCW), infection control measures, preexamination, tracheotomy

## Abstract

*Objective*. To describe coronavirus disease 2019 (COVID-19) patient presentations requiring otolaryngology consultation and provide recommendations for protective measures based on the experience of ear, nose, and throat (ENT) departments in 4 Chinese hospitals during the COVID-19 pandemic.

*Study Design*. Retrospective case series.

*Setting*. Multicenter.

*Subjects and Methods*. Twenty hospitalized COVID-19 patients requiring ENT consultation from 3 designated COVID-19 hospitals in Wuhan, Shanghai, and Shenzhen were identified. Data on demographics, comorbidities, COVID-19 symptoms and severity, consult reason, treatment, and personal protective equipment (PPE) use were collected and analyzed. Infection control strategies implemented for ENT outpatients and emergency room visits at the Eye and ENT Hospital of Fudan University were reported.

*Results*. Median age was 63 years, 55% were male, and 95% were in severe or critical condition. Six tracheotomies were performed. Posttracheotomy outcomes were mixed (2 deaths, 2 patients comatose, all living patients still hospitalized). Other consults included epistaxis, pharyngitis, nasal congestion, hyposmia, rhinitis, otitis externa, dizziness, and tinnitus. At all hospitals, powered air-supply filter respirators (PAPRs) were used for tracheotomy or bleeding control. PAPR or N95-equivalent masks plus full protective clothing were used for other complaints. No inpatient ENT providers were infected. After implementation of infection control strategies for outpatient clinics, emergency visits, and surgeries, no providers were infected at the Eye and ENT Hospital of Fudan University.

*Conclusions and Relevance*. COVID-19 patients require ENT consultation for many reasons, including tracheotomy. Otolaryngologists play an indispensable role in the treatment of COVID-19 patients but, due to their work, are at high risk of exposure. Appropriate protective strategies can prevent infection of otolaryngologists.

Coronavirus disease 2019 (COVID-19) is an infectious respiratory disease caused by the novel virus severe acute respiratory syndrome coronavirus 2 (SARS-CoV-2).^1-4^ As of March 26, 2020, a total of 462,684 cases of COVID-19 have been reported in 199 countries and regions.^5^ On March 11, 2020, the World Health Organization (WHO) declared COVID-19 a global pandemic.^6^

Due to lack of adequate awareness and sufficient infection control plans during the early stages of the pandemic, many health care workers (HCWs) were infected. In particular, HCWs in “noninfectious disease” specialties such as otolaryngology were infected at higher rates than colleagues in the same hospitals.^3,7,8^ According to the data of Italian National Health Agency, 6414 health care workers have been infected with SARS-CoV-2 as of March 26, 2020.^9^

Otolaryngologists are by the nature of their work at high risk for exposure to respiratory pathogens. During the course of routine evaluation and management of patients, otolaryngologists and their staff will inevitably come into direct contact with upper respiratory tract secretions or blood, which may become aerosolized during an inadvertent sneeze or cough.^10^ Such events can occur during a nasal and upper airway endoscopy, while performing a nasal culture or nasopharyngeal swab for SARS-CoV-2, or simply during routine examination of the oral cavity and oropharynx, exposing the health care provider to potentially infectious agents. Clinical data indicate that approximately half of the patients with COVID-19 do not have fever during early stages of the disease, with some patients presenting to the otolaryngologist with fairly innocuous symptoms such as nasal congestion, sore throat, and hyposmia.^3,11-14^ Other patients may present with or be followed for upper aerodigestive track malignancies. These patients commonly complain of cough and sore throat, and coexisting COVID-19 infection may not be considered at presentation. This puts providers and other patients at high risk of infection due to insufficient personal protective equipment (PPE) use unless a high level of clinical suspicion is maintained. In the inpatient setting, critically ill patients with respiratory failure commonly require endotracheal intubation and/or a tracheotomy for respiratory support. In a previous study, 6.30% (15/238) patients required tracheotomy during the severe acute respiratory syndrome (SARS) outbreak in Singapore.^15^ According to clinical statistics, 7.3% to 32% of patients with COVID-19 progress to a severe or critically ill condition, a number of whom may subsequently require tracheotomy for a variety of reasons.^3,4,7,11,16^ Some of these patients may develop complications such as pharyngeal ulcers or bleeding from the tracheotomy site, requiring further ear, nose, and throat (ENT) care. Other emergent events unrelated to COVID-19 but occurring in COVID-19 patients such as epistaxis also require ENT intervention. Given all of these possible routes of exposures, it is critical for ENT departments and providers to establish proper and sufficient infection control measures.

There is little in the literature documenting how ENT departments should approach otolaryngologic diseases in patients infected with SARS-CoV-2 and how to best protect otolaryngologists during the COVID-19 pandemic. It is not clear how to best establish efficient strategies to carefully identify and triage patients and ensure appropriate protection during outpatient and inpatient otolaryngology care. Wuhan was the epicenter of the SARS-CoV-2 outbreak in China, while Shanghai and Shenzhen are large metropolitan centers that were also affected by the pandemic. This article aims to review the otolaryngologist experience treating COVID-19 patients in these cities and to propose appropriate protective measures based on these experiences.

## Methods

### Study Design and Participants

This case series was approved by the Institutional Ethics Committee of The Third People’s Hospital of Shenzhen, Wuhan Chinese and Western Medicine Hospital, and Shanghai Public Health Clinical Center. All COVID-19 patients admitted to Wuhan, Shenzhen, and Shanghai hospitals from January 14, 2020, to March 20, 2020, who received consultation in one of these respective ENT departments were included. Oral consent was obtained from patients. The 3 hospitals—Chinese and Western Medicine Hospital of Tongji Medical College, Huazhong University of Science and Technology (one of the designated hospitals for COVID-19 in Wuhan); Shenzhen Third People’s Hospital (the only designated hospital in Shenzhen); and Shanghai Public Health Clinical Center of Fudan University (the only designated hospital for adults in Shanghai)—are responsible for the treatment for COVID-19 patients as assigned by the government. The Eye and ENT Hospital of Fudan University is responsible for ENT consultation for COVID-19 patients in Shanghai assigned by the government. In response to the pandemic, the Eye and ENT Hospital of Fudan University also quickly implemented a variety of protective strategies for ENT inpatient and outpatient care, including preappointment screening, triaging, restriction of nonurgent clinic visits and surgeries, telemedicine, and proper protective equipment. The infection status and condition of patients and their ENT providers were monitored through March 20, 2020.

### Patient Selection and Management

The subjects involved in this study were COVID-19 patients for whom ENT was consulted for tracheotomy or management of ENT-related symptoms, including epistaxis, nasal congestion, allergic rhinitis, sore throat, hyposmia, dizziness, and tinnitus. Of the COVID-19 inpatients from January 14, 2020, to March 20, 2020 (327 cases in Shanghai, 421 cases in Shenzhen, and 1500 cases in Wuhan Chinese and Western Medicine Hospital), 20 patients met criteria and were included for study.

### Data Collection

Data were obtained from 3 hospitals: Wuhan Chinese and Western Medicine Hospital (12 patients), Shenzhen Third People’s Hospital (6 patients), and Shanghai Public Health Clinical Center (2 patients), which had consultation from the Eye and ENT Hospital of Fudan University. Patients were hospitalized from January 14, 2020, to March 20, 2020. The medical records of patients were analyzed by the research team. Clinical as well as treatment and outcome data were obtained from data collection forms in the electronic medical records. The data were reviewed by a team of trained otolaryngologists. The recorded information included demographic data, comorbidities, severity classification and symptoms of COVID-19, reason for ENT consultation, patient outcomes, provider protection level, and infection of HCWs during ENT consultation. Data from outpatient clinics, emergency room, emergency surgeries, elective surgeries, and telemedicine encounters at the Eye and ENT Hospital of Fudan University were also reviewed.

### Statistical Analyses

A descriptive analysis was performed. Categorical variables were described as frequency rates and percentages, and continuous variables were described using mean, median, and interquartile range (IQR) values.

## Results

### Patient Characteristics

The study population included 20 hospitalized COVID-19 patients seen as consults by otolaryngology. Median age was 63 years (range, 32-72 years), 11 of 20 (55%) were male, and 19 of 20 (95%) were in a severe or critical condition. Of these patients, 7 of 20 (35%) were recommended for a tracheotomy after the consultation. The median age of the tracheotomy group was 65.3 years, and all of them were critically ill with 1 or more chronic diseases such as diabetes, hypertension, and/or coronary artery disease (**Table 1**). Six of 7 patients ultimately underwent a tracheotomy, including 3 surgical tracheotomies (STs) and 3 percutaneous dilational tracheotomies (PDTs). The average intubation time for patients undergoing tracheotomy was 12.6 days. As of March 20, 2020, a total of 2 of 6 (33.3%) of these patients were hospitalized in improving condition, while 4 of 6 (66.7%) did not improve significantly. Three patients underwent PDT for prolonged intubation after being unable to be weaned from ventilation over 10 to 14 days. Of these patients, 2 were still hospitalized at the time of data collection and improving. The third patient presented with severe pneumonia, acute respiratory distress syndrome (ARDS), sepsis, myocarditis, metabolic acidosis with decompensated respiratory acidosis, electrolyte disturbances (hyperkalemia, hyponatremia), and sleep apnea-hypopnea syndrome. This patient was treated with continuous renal replacement therapy (CRRT) and extracorporeal membrane oxygenation (ECMO) accompanied by intra-airway hemorrhage before tracheotomy. Ten days after tracheotomy, the patient experienced bleeding from the nose and mouth. Nineteen days after tracheotomy, obstruction of ECMO flow occurred, and the patient died after recanalization. Another 3 patients underwent ST. The first was a cerebral infarction patient with atrial fibrillation, hypertension, diabetes, cerebral hernia, and anemia who underwent tracheotomy to prevent the occurrence of aspiration pneumonia. He remained in a coma after tracheotomy. Another patient experienced dyspnea after extubation, necessitating an emergent ST rather than reintubation. He was also in a coma as of the date of data collection. The third patient underwent ST because of repeated extubation and reintubation and underwent ST for the second time due to airway bleeding 9 days later. Epistaxis occurred on day 7 after the second tracheotomy, which resolved after temporary packing with nondissolvable packing for 2 days. This patient eventually died on day 10 after the second ST. Overall, outcomes after tracheotomy were mixed with 2 deaths, 2 patients comatose, and all living patients still hospitalized.

**Table 1.** Clinical Presentations of COVID-19 Patients With ENT Symptoms (N = 20).

Clinical presentations	Symptoms consulted by otolaryngologists							
	Tracheotomy	Epistaxis	Pharyngitis	Nasal congestion/hyposmia	Rhinitis	Dizziness and tinnitus	Otitis externa	Total No.
No./total No. (%)	7/20 (35)	6/20 (30)	2/20 (10)	2/20 (10)	1/20 (5)	1/20 (5)	1/20 (5)	20
Age, median, y	65.3	62	67	34	48	52	62	63
Sex, female/male, No.	1/6	4/2	0/2	2/0	1/0	0/1	1/0	9/11
COVID-19 phenotype, No.								
Mild	0	0	0	1	0	0	0	1
Severe	0	5	1	1	1	1	1	10
Critical	7	1	1	0	0	0	0	9
Complaints and symptoms of COVID-19, No.								
Fever	6	2	2	0	1	0	1	12
Cough	3	4	2	1	1	0	1	12
Fatigue	0	1	0	0	0	0	0	1
Shortness of breath	2	0	0	0	0	0	0	2
Chest congestion	1	1	1	0	0	1	0	4
Nasal congestion	0	1	0	1	0	0	0	2
Diarrhea	1	1	0	0	0	0	0	2
Comorbid disorder, No.								
Hypertension	6	2	2	0	0	0	0	4
Diabetes	3	2	0	0	0	1	0	6
Coronary heart disease	2	0	0	0	0	0	0	2
Hyperlipidemia	0	1	1	0	0	0	0	2
Cerebrovascular disease	1	0	0	0	0	0	0	1
COPD	1	0	0	0	0	0	0	1
Valvular heart disease	0	0	1	0	0	0	0	1
Chronic kidney disease	0	1	0	0	0	0	0	1
Ménière’s disease	0	0	0	0	0	1	0	1
Hepatitis B infection	0	0	0	0	0	0	1	1

Abbreviations: COPD, chronic obstructive pulmonary disease; COVID-19, coronavirus disease 2019; ENT, ear, nose, and throat.

Six of the 20 patients (30%) presented with unilateral (3 cases) or bilateral (3 cases) epistaxis. Two patients with bilateral epistaxis had previously been on anticoagulants. Five patients were using noninvasive assisted ventilation or high-flow oxygen through nasal canula, and 1 patient had been treated with ECMO. Their noses were temporarily packed with no further bleeding after removal of their packing 2 days later. No postprocedural complications occurred in the 6 patients except in the 1 patient who was treated with ECMO. He remained in a coma as of data collection.

Of the other 7 patients, 2 were evaluated for sore throats and 2 for nasal congestion (1 of whom also had hyposmia). The last 3 patients presented with symptoms of rhinitis, otitis externa, and tinnitus, respectively. Other than the hyposmia, the symptoms of these 7 patients were alleviated or ameliorated with routine medical treatment (**Table 2**).

**Table 2.** Managements of COVID-19 Patients With Otolaryngologic Complaints.

Complaints and symptoms consulted by otolaryngologists	Therapy for COVID-19 before consultation	Therapy for ENT symptoms	Length of intubation	Complications of treatment	Posttreatment outcome	Medical protection class for ENTs	ENT infection^a^
Tracheotomy (n = 7)^b^							
Prolonged intubation for 16 days, intra-airway bleeding	Intubation, CRRT, ECMO	PDT	16 days	None	Deceased 19 days later	Third level	None
Prolonged intubation for 10 days	High-flow oxygen, intubation	PDT	10 days	None	Improved but still hospitalized	Third level	None
Prolonged intubation for 10 days	Intubation	PDT	10 days	Lung secretions, discharge	Improved but still hospitalized	Third level	None
Prevention of hypostatic pneumonia	None	ST	None	None	Lung infection recovered, comatose	Third level	None
Dyspnea after extubation	High-flow oxygen, noninvasive assisted ventilation, intubation, CRRT	ST	17 days	None	Comatose	Third level	None
Repeated extubation, then intra-airway bleeding	Noninvasive assisted ventilation, intubation, ECMO	ST (×2)	10 days	Intra-airway hemorrhage on day 9 after the first ST, epistaxis occurred on day 7 after the second tracheotomy	Died of cardiac arrest during a lung transplant 10 days after the second ST	Third level	None
Epistaxis (n = 6)							
Unilateral (3)	Noninvasive assisted ventilation (2), nasal high-flow oxygen (1)	Hemostasis with packing	None	Hemoptysis (3)	Resolution of epistaxis (3)	Third level	None
Bilateral (3)	Noninvasive assisted ventilation (1), nasal high-flow oxygen (1)	Hemostasis with packing	None	Hemoptysis (2)	Resolution of epistaxis (2)	Third level	None
	ECMO (1)	Hemostasis with packing	None	None	Resolution of epistaxis, comatose (1)	Third level	None
Pharyngitis (n = 2)	Noninvasive assisted ventilation (2), CRRT (1), ECMO (1)	Compound borax mouthwash (1), compound chlorhexidine mouthwash (1)	None	Pharyngeal reflex (1)	Symptoms alleviated (2)	Third level (Wuhan)/second level (Shenzhen)	None
Nasal congestion (n = 1)	None	Oxymetazoline nasal spray	None	Sneeze	Symptoms alleviated	Third level (Wuhan)	None
Nasal congestion and hyposmia (n = 1)	None	Renault Court nasal spray	None	None	Nasal congestion alleviated, hyposmia not alleviated	Second level (Shenzhen)	None
Rhinitis (n = 1)	Nasal high-flow oxygen	Fluticasone propionate nasal spray	None	Sneeze	Symptoms alleviated	Third level (Wuhan)	None
Dizziness and tinnitus (n = 1)	Nasal high-flow oxygen	Betahistine in remission	None	None	Symptoms alleviated	Third level (Wuhan)	None
Otitis externa (n = 1)	Noninvasive assisted ventilation	Mupirocin	None	None	Symptoms alleviated	Third level (Wuhan)	None

Abbreviations: COVID-19, coronavirus disease 2019; CRRT, continuous renal replacement therapy; ECMO, extracorporeal membrane oxygenation; ENT, ear, nose, and throat department; PDT, percutaneous dilational tracheostomy; ST, surgical tracheostomy.

a Health care workers (HCWs) in Wuhan and Shenzhen took their temperature 4 times every day and took nucleic acid tests twice spanning a 24-hour period. HCWs in Shanghai took their temperature twice every day and took nucleic acid tests on days 7, 12, and 14.

b One patient declined tracheotomy after comprehensive assessment of the patient’s condition.

### Infectious Control in HCWs

**Figure 1.**
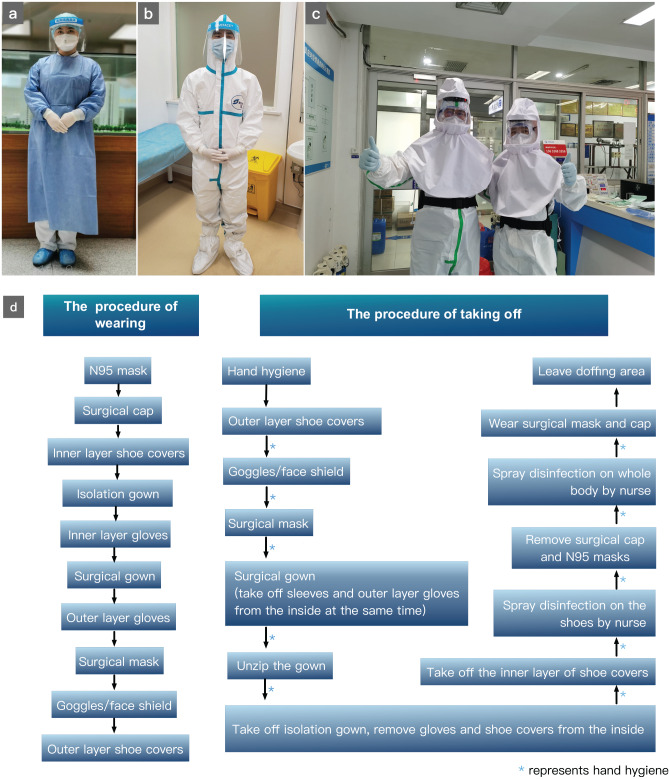
Medical protection of health care workers. Example of provider wearing first-level (a), second-level (b), and third-level (c) protection. (d) Protocol for donning and doffing personal protective equipment.

When treating patients for common otolaryngologic diagnoses or symptoms such as rhinitis, nasal congestion, hyposmia, sore throat, dizziness, tinnitus, and otitis externa, the otolaryngology HCWs in the epidemic center (Wuhan) and other areas (Shenzhen, Shanghai) took third-level protection and second-level protection, respectively (**Table 3** and **Figure 1**). The second-level protective measures include wearing medical protective masks equivalent to N95 respirators, eye protection such as goggles or face shields, work clothes, disposable isolation gowns and/or coveralls, shoe covers, gloves, and hair covers. The third-level protection measures added a powered air-supply filter respirator (PAPR) such as a positive pressure headgear or a comprehensive respiratory protective device according to expert consensus in China.^17^

**Table 3.** Medical Protection for Otolaryngologists.^a^

Medical protection class for HCWs	Application or occasion	Normal surgical mask	Medical protective mask^b^	Goggles/face shield	PAPR	Work clothes	Coverall (protective clothing)	Isolation gowns	Gloves	Shoe covers	Hair cover
First level	Preexamination and triage of outpatients^c^	•	○			•		○	○		•
	Normal outpatient, emergency, ward^c^	•	○	○		•		○	•		•
	Touring or cleaning in potentially contaminated area outside isolation ward		•			•		•	•	•	•
	Intubation, tracheostomy nasal/laryngeal endoscopy for ordinary patients		•	○		•		•	•		•
	Surgery on ordinary patients^c,d^	•	○			•			•		•
	Medical observation area for HCWs	•				•					•
	Close contact of tracheotomy^e,f^		•	○		•	○	•			•
Second level	Normal consultation, physical examination, nursing or sampling in isolation ward^f^		•	•		•	•	○	•	•	•
	Transport or accompanying examination of suspected or confirmed patients		•	•		•	•		•	•	•
Third level	Intubation, tracheostomy, nasal/laryngeal endoscopy for suspected or confirmed patients^g^		•	•	○	•	•		•	•	•
	Surgery on suspected or confirmed patients^c,g,h^		•	•	○	•	•	○	•	•	•

Abbreviations: HCW, health care worker; PAPR, powered air-purifying respirator

a • indicates priority selection; ○ indicates selection if necessary. Blanks cells indicate not selected.

b The same or higher level of protective masks as N95 masks.

c Medical protective masks and normal surgical masks are not used at the same time.

d The work clothes here are scrubs.

e Protective clothing or isolation gowns could be used according to the possibility of infection after close contacts.

f Generally, the isolation gowns and protective clothing are not used at the same time. Some operations may contaminate protective clothing. For nursing or when moving between different isolation patients, it is recommended to use isolation gowns in addition to protective clothing; when in close contact with a tracheostomy, protective clothing or isolation gowns could be used according to the possibility of infection after close contacts.

g If PAPR is selected, medical protective masks and goggles/face shield are not used.

h Wear an isolation gown if necessary (when prone to contamination).

Otolaryngologists performing a tracheotomy or procedures for control of hemorrhage in circumstances of tracheal/oral bleeding or epistaxis in patients infected with SARS-CoV-2 used third-level infection control measures whether in Wuhan, Shenzhen, or Shanghai. Importantly, proper technique for donning and doffing PPE must be practiced to effectively protect otolaryngology providers (**Figure 1**). Under the guidance of trained staff in the isolation ward, providers disposed of the waste and sharps properly and disinfected personal supplies brought out from the contaminated area after the procedures. Outer protective equipment was removed at the entrance of the semicontaminated area. The inner protective equipment was removed in the semicontaminated area and personal supplies were disinfected again.^18^ Furthermore, careful attention was given to proper hand hygiene and avoiding contact with the eyes, nose, and mouth. The otolaryngology health care worker was then quarantined in designated isolation locations for 14 days after the consultation. Health care workers in Wuhan and Shenzhen took their temperature 4 times every day and took nucleic acid tests twice spanning a 24-hour period. HCWs in Shanghai took their temperature 2 times every day and took nucleic acid tests on days 7, 12, and 14, a total of 3 times. At the time of data collection, no otolaryngologists who participated in the care of the patients in this study had become infected with SARS-CoV-2.

**Figure 2.**
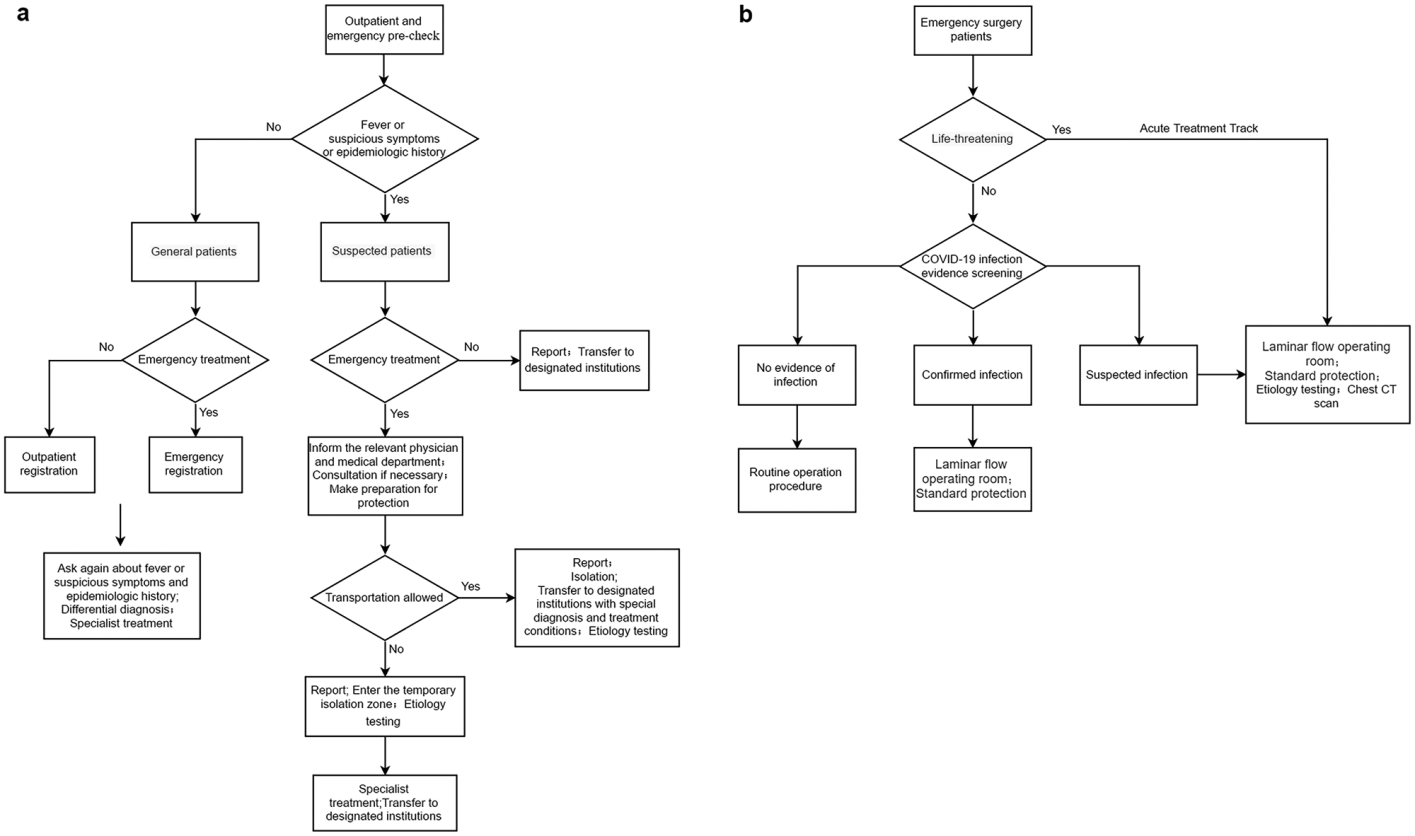
Algorithm for triage of patients during the coronavirus disease 2019 pandemic. (a) Recommended precheck and triage algorithm for outpatient clinics and emergency room. (b) Recommended algorithm for preoperative triage.

The 3 hospitals in Wuhan, Shenzhen, and Shanghai were designated for treatment of COVID-19 patients, and so their outpatient clinics and emergency departments were closed. However, the Eye and ENT Hospital of Fudan University cares for one of the largest numbers of ENT patients in China and continued to do so throughout the pandemic. Since the announcement of obvious human-to-human transmission on January 20, 2020, the Eye and ENT Hospital of Fudan University reduced its large number of outpatient visits immediately by eliminating nonurgent visits, stopped elective surgery, avoided upper aerodigestive tract endoscopic examinations as much as possible, developed outpatient and emergency room triage flowcharts (**Figure 2**), and improved staff training on infection control measures. In the preexamination areas for the emergency rooms, outpatient clinics, and inpatient wards, HCWs used first-level protection measures consisting of work clothes, isolation gowns, hair cover, normal surgical masks, and gloves when necessary. Second-level protection was used in the isolation ward and third-level protection in the operating room and when performing invasive procedures. The protection level can be adjusted according to the specific type of encounter and working areas (see **Table 3** for details). We developed and followed these procedures and then gradually increased outpatient appointments, tumor-related surgery, and then finally open elective surgery according to the pandemic situation. At present, our clinic and surgical volume has returned to baseline. One-fourth as many outpatient visits and one-sixth as many elective surgeries were performed from January 20, 2020, to March 20, 2020 compared to the same period of 2019. In lieu of normal patient visits, 5765 telemedicine encounters were performed. No further HCW or patient infections have occurred.

## Discussion

In this clinical study of 20 COVID-19 patients seeking ENT consultation, 5 patients who had previously been endotracheally intubated were converted to a tracheotomy and 1 patient underwent a tracheotomy only. Otolaryngologists performed a tracheotomy for these patients for management of secretions, repeated endotracheal intubation, and long-term endotracheal intubation. Data from non-COVID-19 infected critically ill patients suggest that early tracheotomy (within 10 days of intubation) has been associated with more ventilator-free days, shorter intensive care unit (ICU) stays, shorter duration of sedation, and lower long-term mortality rates, although other studies have found that timing of tracheotomy does not affect important clinical outcomes.^19,20^ In this study, 4 of the 6 patients (66.7%) who underwent tracheotomy subsequently were in a coma (2 cases) or died (2 cases), which underscores the fact that in this small patient cohort, most patients did not achieve clinical benefit from the tracheotomy performed. Another report from China also suggests against benefit from tracheotomy for COVID-19 patients.^21^ There are also data from the SARS treatment experience suggesting that tracheotomy was not associated with significant improved outcome.^22^ Tracheotomy is also associated with a number of potential complications, including tracheal bleeding. Without specific treatment for the infection, the mortality rate once a patient goes into severe or critical ARDS is as high as 70%, which also may argue against proceeding with tracheostomy on patients with COVID-related ARDS.^23^ Taken in combination with our clinical experience in China, we recommend that long-term intubation should not on its own be an indication for tracheotomy in COVID-19 patients, as the risk to patients and providers likely outweighs any marginal benefits in this scenario. Rather, tracheotomy should only be used in specific situations such as airway obstruction where the potential for successful extubation is otherwise compromised or in circumstances where tracheotomy placement might positively affect a patient’s potential for successful weaning of minimal ventilatory support. These scenarios do require careful consideration when medical resources including ventilators are in limited supply in the setting of widespread community infection. In agreement with recent recommendations released by ENT UK, we believe such clinical situations should be evaluated in a multidisciplinary fashion such that consensus among specialists regarding potential for clinical benefit following tracheotomy as weighed against the risk of the procedure is agreed upon prior to proceeding with the procedure.^24^

In this study, 30% of consults were for epistaxis. This finding may be associated with the patients breathing dry cold air (due to hospitals turning off heating systems to minimize airborne transmission), long-term oxygen inhalation, using anticoagulants, acquired coagulopathies secondary to severe illness, and comorbidities such as hypertension or diabetes. Given that the SARS-CoV-2 viral load has been found to be highest in the nose, any intervention involving the nasal cavity—particularly those that could produce aerosolized blood or secretions—should be considered high risk, and appropriate PPE should be used accordingly.^25^

SARS-CoV-2 is very infectious, with a control reproduction number that may be as high as 6.47 (95% CI, 5.71-7.23), which is higher than the SARS transmission number in 2003.^26,27^ As previously discussed, ENT providers may be subjected to high-risk exposures in a variety of different scenarios. For this reason, it is important that health workers in ENT departments use standard precautions consistently when providing care to COVID-19 patients. Rigorous implementation and adherence are crucial for the control of outbreak situations. Based on our previous experience of performing tracheotomy during SARS, no medical or nursing staff member was infected after carrying out the procedure while taking all the precautions and wearing the appropriate protective apparel.^15,22,28,29^ Medical units should provide graded protection for health care workers according to the job position, work area, and the level of exposure risk.^17^ PPE is the most obvious aspect of infection control. PPE must be correctly selected and used in a safe manner. Based on expert consensus in China, HCWs adopted third-level protection measures when performing invasive procedures such as tracheotomy or control of bleeding. The second-level protection measures can be taken for evaluation, treatment, or throat swab sampling of patients in the isolation ward. The utilization of PPE in second-level protection and third-level protection here roughly corresponds to standard PPE and enhanced PPE, respectively, as previously described by the Centers for Disease Control and Prevention (CDC).^15,29^ Wuhan was the epicenter of the pandemic, with a higher incidence of disease than Shenzhen and Shanghai. As such, during consultation of COVID-19 patients with sore throat and nasal congestion, otolaryngologists in Wuhan and Shenzhen adopted third-level and secondary-level infection control plans, respectively, with no resulting provider infections at either hospital. These clinical results suggest that the adoption of such graded protection measures is feasible and effective. Health care workers could choose different-level protection according to disease prevalence, the degree of exposure risk, and the availability of PPE. While in this study all otolaryngology providers were quarantined for 14 days after caring for COVID-19 positive patients, the ultimate lack of infection of any of these providers may indicate that such quarantine is not necessary if appropriate PPE and other infection control measures are used. Serial temperature checks and viral testing could be used instead to confirm providers are not infected without having to remove them from the workforce, a key concern given the potential volume of COVID-19 patients requiring care. Further study is necessary to determine what is the minimum sufficient level of PPE to prevent infection with COVID in different clinical scenarios.

It is not always possible to identify patients with SARS-CoV-2 infection on clinical presentation alone as early symptoms are nonspecific. This can lead to high-risk exposures in the outpatient setting if high clinical suspicion is not maintained. In 1 example, a patient who was seen in the otolaryngology department for a sore throat and mild cough was subsequently diagnosed with COVID-19, illustrating the importance of careful triage of outpatients.^30^ Some evidence also indicates that hyposmia or loss of smell may be an early symptom of SARS-CoV-2 infection, which may be particularly relevant to otolaryngologists.^13,14^ In our cohort, there was at least 1 case of a mild severity patient who initially presented to an otolaryngologist for hyposmia. At the Eye and ENT Hospital of Fudan University, proactive implementation of a variety of protective strategies immediately at the start of the outbreak resulted in no HCW infections occurring despite high exposure risk, indicating the effectiveness of these policies. Notably, as the overall prevalence of disease stabilized, the hospital was able to increase its clinical and surgical volume back to baseline while still having no HCW infections. While this is in part a reflection of the success of national disease control measures in China, it also suggests that with proper patient screening, protective equipment, and training, even a high-risk specialty such as otolaryngology can practice safely and effectively in the midst of the COVID-19 pandemic.

There were some limitations to our study. As with all retrospective studies, the conclusions that can be drawn are limited by the observational and heterogenous nature of the data. There are also only 20 subjects included in this study. As such, this may not fully display the clinical characteristics of COVID-19 patients requiring ENT consultation. It also limits the conclusions that can be drawn regarding the feasibility and effectiveness of the protective measures taken by the otolaryngology providers participating in the diagnosis and treatment.

## Conclusions

We found that COVID-19 positive inpatients can require intervention by an otolaryngologist for a variety of reasons, of which a tracheotomy was the most common in this study. Otolaryngologists play an indispensable role in the treatment of COVID-19 patients, but due to the inherent nature of their work, they are at high risk of exposure whether working in outpatient clinics, the emergency room, or inpatient wards. Appropriate use of PPE titrated to the level of exposure can prevent SARS-CoV-2 infection among otolaryngologists. Establishment of strategies of caring for general ENT patients during the COVID-19 outbreak can protect health care workers.

## References

[1] Zhou P Yang X-L Wang X-G, et al A pneumonia outbreak associated with a new coronavirus of probable bat origin [published online 2 3, 2020]. Nature. 10.1038/s41586-020-2012-7 PMC7095418 32015507

[2] Wu F Zhao S Yu B, et al A new coronavirus associated with human respiratory disease in China [published online 2 3, 2020]. Nature. 10.1038/s41586-020-2008-3 PMC7094943 32015508

[3] Wang D Hu B Hu C, et al Clinical characteristics of 138 hospitalized patients with 2019 novel coronavirus-infected pneumonia in Wuhan, China [published online 2 7, 2020]. JAMA. 10.1001/jama.2020.1585 PMC7042881 32031570

[4] Huang C Wang Y Li X, et al Clinical features of patients infected with 2019 novel coronavirus in Wuhan, China. Lancet. 2020;395(10223):497-506. 31986264 10.1016/S0140-6736(20)30183-5 PMC7159299

[5] World Health Organization. Coronavirus disease 2019 (COVID-19) situation report–66. Published 3 26, 2020 Accessed March 27, 2020 https://www.who.int/docs/default-source/coronaviruse/situation-reports/20200326-sitrep-66-covid-19.pdf?sfvrsn = 81b94e61_2

[6] World Health Organization. WHO director-general’s opening remarks at the media briefing on COVID-19. Published 3 11, 2020 Accessed March 27, 2020 https://www.who.int/dg/speeches/detail/who-director-general-s-opening-remarks-at-the-media-briefing-on-covid-19—11-march-2020

[7] McGoogan ZWJM Characteristics of and important lessons from the coronavirus disease 2019 (COVID-19) outbreak in China: summary of a report of 72314 cases from the Chinese Center for Disease Control and Prevention. JAMA. 2020;323(13):1239-1242. 10.1001/jama.2020.2648 32091533

[8] Patel ZM Fernandez-Miranda J Hwang PH, et al Precautions for endoscopic transnasal skull base surgery during the COVID-19 pandemic [published online 3 19, 2020]. Neurosurgery. 10.1093/neuros/nyaa125 PMC7184431 32293678

[9] The COVID-19 Task force of the Department of Infectious Diseases and the IT Service Istituto Superiore di Sanità. Integrated surveillance of COVID-19 in Italy. Published 3 26, 2020 Accessed March 27, 2020 https://www.epicentro.iss.it/coronavirus/bollettino/Infografica_26marzo%20ENG.pdf

[10] Tran K Cimon K Severn M Pessoa-Silva CL Conly J. Aerosol generating procedures and risk of transmission of acute respiratory infections to healthcare workers: a systematic review. PLoS ONE. 2012;7(4):e35797. 22563403 10.1371/journal.pone.0035797 PMC3338532

[11] Guan WJ Ni ZY Hu Y, et al Clinical characteristics of coronavirus disease 2019 in China [published online 2 28, 2020]. N Engl J Med. 10.1056/NEJMoa2002032 PMC7092819 32109013

[12] Chen N Zhou M Dong X, et al Epidemiological and clinical characteristics of 99 cases of 2019 novel coronavirus pneumonia in Wuhan, China: a descriptive study [published online 1 30, 2020]. Lancet. 10.1016/S0140-6736(20)30211-7 PMC7135076 32007143

[13] Mao L Wang M Chen S, et al Neurological manifestations of hospitalized patients with COVID-19 in Wuhan, China: a retrospective case series study. medRxiv. 2020.

[14] Baig AM Khaleeq A Ali U Syeda H. Evidence of the COVID-19 virus targeting the CNS: tissue distribution, host-virus interaction, and proposed neurotropic mechanisms. ACS Chem Neurosci. 2020;11(7):995-998. 32167747 10.1021/acschemneuro.0c00122

[15] Chee VW Khoo ML Lee SF Lai YC Chin NM. Infection control measures for operative procedures in severe acute respiratory syndrome-related patients. Anesthesiology. 2004;100(6):1394-1398. 10.1097/00000542-200406000-00010 15166557

[16] Yang X Yu Y Xu J, et al Clinical course and outcomes of critically ill patients with SARS-CoV-2 pneumonia in Wuhan, China: a single-centered, retrospective, observational study [published online 2 24, 2020]. Lancet Respir Med. 10.1016/S2213-2600(20)30079-5 PMC7102538 32105632

[17] Chun-hui L Xun H Meng C, et al Expert consensus on personal protection in different regional posts of medical institutions during novel coronavirus pneumonia (COVID-19) epidemic period. Chin J Infect Control. 2020;19(3):1-15.

[18] Ke-lei G Yong L Chang-ning X Wei-hong J Dong-hai H. Summary of the indications and suggestions on the protective measures of tracheotomy during the outbreak of novel coronavirus pneumonia. Chin J Otorhinolaryngol Skull Base Surg. 2020;26(1):9-13.

[19] Hosokawa K Nishimura M Egi M Vincent JL. Timing of tracheotomy in ICU patients: a systematic review of randomized controlled trials. Crit Care. 2015;19:424. 26635016 10.1186/s13054-015-1138-8 PMC4669624

[20] Wang F Wu Y Bo L, et al The timing of tracheotomy in critically ill patients undergoing mechanical ventilation: a systematic review and meta-analysis of randomized controlled trials. Chest. 2011;140(6):1456-1465. 21940770 10.1378/chest.11-2024

[21] Yu S Yujuan H Hongjun X. Recommendations for diagnosis and treatment of emergency diseases in ENT surgery during the prevention and control of new coronavirus. Chin J Otorhinolaryngol Head Neck Surg. 2020(04):322-325

[22] Wei WI Tuen HH Ng RW Lam LK. Safe tracheostomy for patients with severe acute respiratory syndrome. Laryngoscope. 2003;113(10):1777-1779. 14520105 10.1097/00005537-200310000-00022 PMC7165919

[23] Liu Y Sun W Li J, et al Clinical features and progression of acute respiratory distress syndrome in 1 coronavirus disease 2019. medRxiv. 2020.

[24] ENT UK. Guidance for surgical tracheostomy and tracheostomy tube change during the COVID-19 pandemic. Accessed March 27, 2020 https://www.entuk.org/tracheostomy-guidance-during-covid-19-pandemic 10.1007/s12070-020-01893-y PMC7306934 32719738

[25] Zou L Ruan F Huang M, et al SARS-CoV-2 viral load in upper respiratory specimens of infected patients [published online 3 19, 2020]. N Engl J Med. 10.1056/NEJMc2001737 PMC7121626 32074444

[26] Tang B Wang X Li Q, et al Estimation of the transmission risk of the 2019-nCoV and its implication for public health interventions. J Clin Med. 2020;9(2):462. 10.3390/jcm9020462 PMC7074281 32046137

[27] Bauch CT Lloyd-Smith JO Coffee MP Galvani AP. Dynamically modeling SARS and other newly emerging respiratory illnesses: past, present, and future. Epidemiology. 2005;16(6):791-801. 16222170 10.1097/01.ede.0000181633.80269.4c

[28] Yam LY Chen RC Zhong NS. SARS: ventilatory and intensive care. Respirology. 2003;8(suppl):S31-S35. 15018131 10.1046/j.1440-1843.2003.00521.x PMC7169203

[29] Tien HC Chughtai T Jogeklar A Cooper AB Brenneman F. Elective and emergency surgery in patients with severe acute respiratory syndrome (SARS). Can J Surg. 2005;48(1):71-74. 15757044 PMC3211568

[30] Zhang K Zhao Y Cui C, et al Suggestions for early identification of 2019 novel coronavirus infection in otolaryngology head and neck surgery [in Chinese]. Chin J Otorhinolaryngol Head Neck Surg. 2020;55:E001. 10.3760/cma.j.cn115330-20200213-00082 33342133

